# Activity-Dependent Plasticity of Axo-axonic Synapses at the Axon Initial Segment

**DOI:** 10.1016/j.neuron.2020.01.037

**Published:** 2020-04-22

**Authors:** Alejandro Pan-Vazquez, Winnie Wefelmeyer, Victoria Gonzalez Sabater, Guilherme Neves, Juan Burrone

**Affiliations:** 1 MRC Centre for Neurodevelopmental Disorders, Institute of Psychiatry, Psychology and Neuroscience, King’s College London, London SE1 1UL, UK; 2 Centre for Developmental Neurobiology, Institute of Psychiatry, Psychology and Neuroscience, King’s College London, London SE1 1UL, UK; 3 The Rosalind Franklin Institute, Harwell Campus, Didcot OX11 0FA, UK

**Keywords:** Chandelier, axo-axonic, homeostatic, interneuron, plasticity, GABA, activity-dependent, axon initial segment, development

## Abstract

The activity-dependent rules that govern the wiring of GABAergic interneurons are not well understood. Chandelier cells (ChCs) are a type of GABAergic interneuron that control pyramidal cell output through axo-axonic synapses that target the axon initial segment. *In vivo* imaging of ChCs during development uncovered a narrow window (P12–P18) over which axons arborized and formed connections. We found that increases in the activity of either pyramidal cells or individual ChCs during this temporal window result in a reversible decrease in axo-axonic connections. Voltage imaging of GABAergic transmission at the axon initial segment (AIS) showed that axo-axonic synapses were depolarizing during this period. Identical manipulations of network activity in older mice (P40–P46), when ChC synapses are inhibitory, resulted instead in an increase in axo-axonic synapses. We propose that the direction of ChC synaptic plasticity follows homeostatic rules that depend on the polarity of axo-axonic synapses.

## Highlights

• Axo-axonic synapses and Chandelier cell axons develop rapidly between P12 and P18
• Increasing network activity during this period reduces axo-axonic synapse number
• The depolarizing nature of axo-axonic synapses suggests this plasticity is homeostatic
• When axo-axonic synapses become hyperpolarizing, synapse plasticity is reversed

## Teaser

Pan-Vazquez et al. investigate the development and plasticity of axo-axonic synapses formed by Chandelier cell interneurons. They find that these synapses follow homeostatic plasticity rules, where the direction of plasticity changes together with the developmental switch in GABA polarity.

## Introduction

An important question in neuroscience is how neurons in the brain wire up and adapt their connectivity to form functional circuits. In the cortex, circuits are typically formed of excitatory pyramidal neurons and GABAergic interneurons that modulate the activity of pyramidal cells through local connections. Whereas many studies have focused on the emergence of excitatory synapses, the rules that govern the formation of GABAergic synapses are less well understood (De Marco García et al., 2011, Favuzzi et al., 2019, Karayannis et al., 2012), despite their importance in circuit function (Kepecs and Fishell, 2014). Among the highly heterogeneous population of GABAergic interneurons that provide inhibition in the cortex, Chandelier cells (ChCs) are one of the least well characterized, mainly due to their sparseness and the lack of genetic tools to label them until recently (Taniguchi et al., 2013). Yet, they display many morphological features that make them particularly good candidates for controlling both neuronal and circuit activity in the brain (Inan et al., 2013, Somogyi, 1977). Although sparsely distributed throughout the cortex, ChC axons are highly ramified, resulting in the dense innervation of hundreds of neighboring pyramidal neurons, within well-defined cortical domains (Inan et al., 2013). A salient feature of the ChC axon is its specific targeting of the axon initial segment (AIS) on pyramidal cells (Peters et al., 1982, Somogyi et al., 1982), a subcellular domain that is responsible for the initiation of action potentials and which is home to unique forms of homeostatic plasticity (Grubb and Burrone, 2010, Kuba et al., 2010, Wefelmeyer et al., 2015, Wefelmeyer et al., 2016). ChCs are therefore thought to exert a powerful influence on neuronal excitability, by providing direct modulation of pyramidal cell output. Indeed, abnormalities in axo-axonic synapses have been associated with developmental brain disorders such as schizophrenia (Fazzari et al., 2010, Lewis, 2011) and epilepsy (DeFelipe, 1999, Marco and DeFelipe, 1997).

Recent work has begun to shed some light on the developmental profile and wiring logic of ChCs in the cortex. The general emerging picture is that, compared to other interneurons, ChCs are delayed in all aspects of their development. Following a relatively late birth and migration into the cortex, ChC axons ramify to form stable cartridges at the AIS around the third postnatal week (Steinecke et al., 2017, Taniguchi et al., 2013), later than the contacts formed by other cortical interneurons (Favuzzi et al., 2019). The formation of these contacts relies on the expression of a specific cell adhesion molecule (L1CAM), which increases its expression levels during this period and is required for the formation and maintenance of axo-axonic contacts at the AIS (Tai et al., 2019). In addition, a recent study has shown a remarkable selectivity in the choice of postsynaptic partners, where L2/3 ChCs in the prefrontal cortex preferentially target pyramidal neurons that project to deeper nuclei such as the basolateral amygdala, rather than those that project to the contralateral hemisphere (Lu et al., 2017). However, although we have a basic understanding of ChC development, we do not yet understand the activity-dependent rules that mold its distinctive wiring pattern.

To date, many basic aspects of ChC physiology remain a mystery. In fact, it is still unclear how ChCs modulate neuronal output at the single-cell level. Previous work has proposed both inhibitory and excitatory (Szabadics et al., 2006, Woodruff et al., 2009, Woodruff et al., 2010) roles for ChC function, which may depend on the state of the network (Woodruff et al., 2011). More recent findings have attempted to reconcile these differences by showing that the developmental stage of the brain defines the polarity of axo-axonic synapses. Building on the notion that GABA switches from being depolarizing to hyperpolarizing during development (Ben-Ari et al., 2007), the effect of GABA released from ChCs appears to follow a similar, albeit delayed, trajectory. Whereas postsynaptic responses elicited by most GABAergic interneurons switch polarity during the first 2 postnatal weeks, ChCs in the prelimbic cortex continue to be depolarizing beyond the third postnatal week (Rinetti-Vargas et al., 2017), resulting in a developmental window where different interneuron types may modulate pyramidal cell activity in opposite directions. Although the transition in GABA polarity is a developmental milestone in circuit function, our understanding of the rules of interneuron plasticity across this switch in polarity remains incomplete. This question is all the more important when we consider that the switch in polarity of ChCs occurs in peri-adolescence (Rinetti-Vargas et al., 2017), right after the period of circuit wiring, a time where activity-dependent plasticity is thought to play an important role in the formation of stable cortical circuits (Andreae and Burrone, 2014, Tien and Kerschensteiner, 2018).

Here, we set out to uncover the rules that govern the development and plasticity of ChC axo-axonic synapses in the mouse cortex. We find that both axo-axonic synapses and their target, the AIS, are highly plastic structures, especially during development. Specifically, we followed the plasticity rules behind ChC connectivity across the developmental switch in GABA polarity. We show that axo-axonic synapse plasticity opposes the changes in the activity of the network, consistent with a model where the wiring of GABAergic synapses follows homeostatic rules that serve to stabilize brain activity, especially during development.

## Results

### Morphological and Electrophysiological Development of ChCs

**Figure 1:**
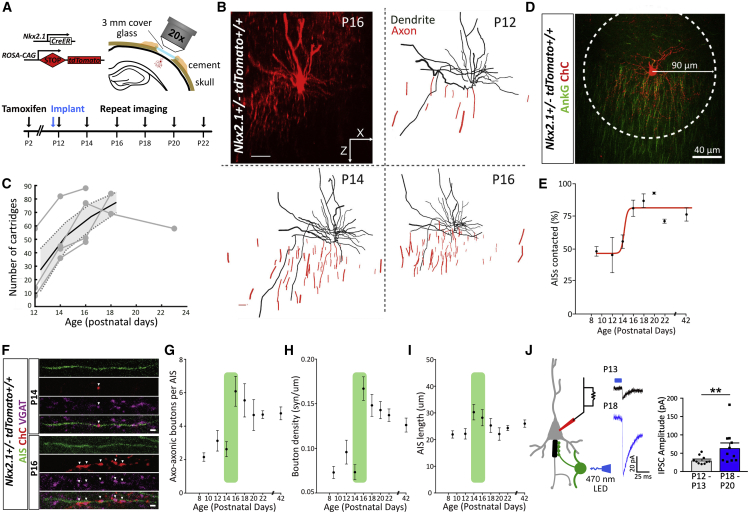
Development of Chandelier Cells and Axo-axonic Synapses in Somatosensory Cortex (A) Genetic strategy and timeline for tamoxifen injection for labeling ChCs in Nkx2.1-CreER^+/−^;Ai9 mice, cranial window implantation, and repeated *in vivo* imaging. (B) *In vivo* image (P16) and reconstructions (P12–P16) of a ChC. Scale bar, 40 μm. (C) Number of cartridges for individual ChCs during development (gray) and mean cartridge number (black, n = 4 ChCs, 3 mice). (D) Image of a ChC (red) and AISs (green) at P18. Connection probability was defined as the percentage of AISs with ChC overlap within a 90 μm radius (white circle). (E) Average connection probability of ChCs across development (4–5 ChCs, 2–4 mice per time point), with a sigmoidal fit (red). (F) Images of axo-axonic synapses located on an AIS and expressing VGAT at P14 and P16. Scale bar, 2 μm. (G–I) Average number (G) and density (H) of axo-axonic boutons as well as (I) AIS length across development, from fixed tissue samples (n = 26–81 AISs, 2–4 mice per time point). The green shaded area highlights the period of rapid synaptic development. (J) ChCs expressing ChR2 were stimulated with light and GABAergic PSCs recorded in nearby pyramidal cells (left). Example responses (middle) and average GABAergic PSC amplitude (right) in immature and mature networks (^∗∗^p < 0.01, Mann-Whitney test. n = 10–11 neurons, 4 mice, per condition). Plots show mean ± SEM. See also Figure S1.

We used a transgenic mouse line (Nkx2.1-CreER) (Taniguchi et al., 2013) crossed with a reporter line (Ai9) to image the development of ChC axons in layer 2/3 of the somatosensory cortex *in vivo* (Figure 1A). This line labels ChCs sparsely, allowing accurate morphological reconstructions of dendritic and axonal arbors. In line with the late arrival of ChCs to the cortex, *in vivo* imaging of individual ChCs over many days also showed a delayed period of axonal growth that peaked within a narrow window across different cells, from P12 to P18. More surprising was the fact that the axonal arbors of individual ChCs showed a rapid transition in their morphology, generally within 2 days, from an immature state with few cartridges, to a highly complex arbor with multiple cartridges that span a well-defined cortical domain (Figures 1B and 1C). The dendrites, on the other hand, appear to develop earlier and remain largely unchanged throughout this period. Mirroring the rapid growth of the axonal arbor, imaging of fixed brain slices at different developmental periods showed that the number of postsynaptic pyramidal cells contacted by an individual ChC also increased during this window (Figures 1D and 1E). Although our data revealed that some connections between ChCs and pyramidal cells existed before P12 (∼50% connectivity on average; Figure 1E), these connections were generally weak, involving few synapses (Figures 1F–1H). Indeed, we observed an abrupt increase in the number of synapses formed onto an AIS (Figures 1F–1H) that followed the increase in axon arbor size, without any changes in AIS length (Figure 1I). In agreement with these morphological findings, we also saw a functional increase in the amplitude (Figure 1J) of GABAergic PSCs recorded from pyramidal cells in response to optogenetic stimulation of ChCs (Figures S1A–S1C) during the period of synaptogenesis, as well as a maturation of the intrinsic firing properties of ChCs themselves (Figures S1D–S1F). We conclude that there is a narrow developmental window (P12–P18) over which ChCs connect to neighboring pyramidal cells and establish a local microcircuit.

### Activity-Dependent Plasticity of Axo-axonic Synapses during Development

**Figure 2:**
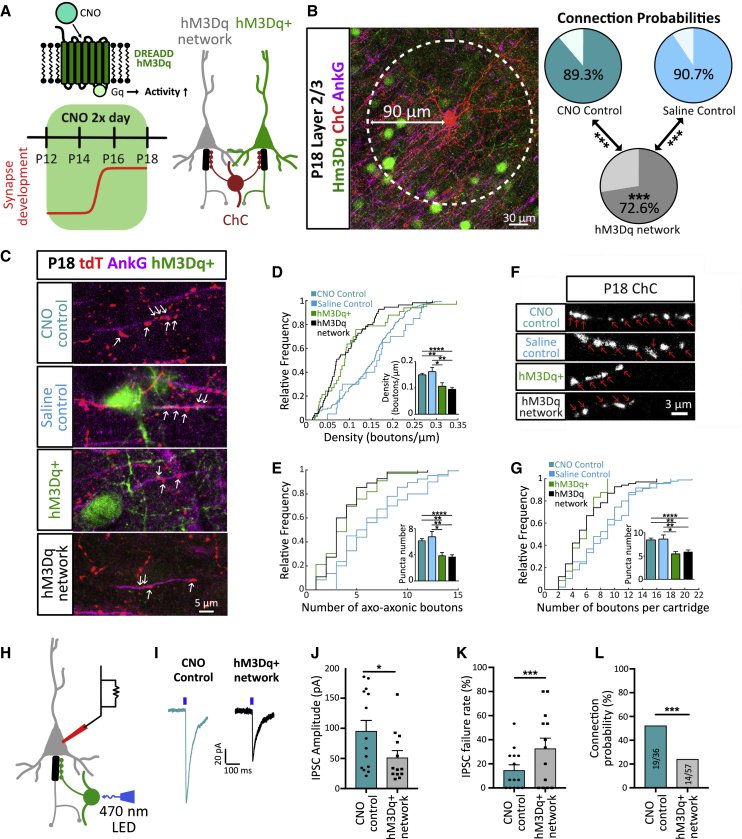
Activity-Dependent Plasticity of Axo-axonic Synapses (A) Schematic of DREADD receptor hM3Dq (top left), timeline of CNO application (below), and logic of experimental design resulting in ChCs contacting hM3Dq^+^ (green) and hM3Dq^–^ (gray) pyramidal cells in the same network. (B) Connection probability of ChCs at P18 in a 90 μm radius (white circle) following injection of CNO into control mice (CNO control), into hM3Dq expressing mice (hM3Dq network) or saline into hM3Dq expressing mice (saline control) (chi-square test. n = 270–390 cells, 3–4 mice, per condition). (C) Example images showing axo-axonic boutons overlapping with the AIS at P18. (D and E) Cumulative distribution of and average axo-axonic bouton density (D) and number (E) (one-way Kruskal-Wallis with Dunn’s multiple comparison test. n = 20–132 cells, 3–4 mice, per condition). (F) Example images of axo-axonic cartridges. (G) Cumulative distribution and average cartridge size (Kruskal-Wallis with Dunn’s multiple comparison test, n = 21–40 neurons, 3–4 mice, per condition). (H and I) Schematic diagram of patch-clamp experiment (H) and representative GABAergic PSCs (I) from pyramidal cells after optogenetic ChC stimulation for CNO control (blue) and hM3Dq-network (black) conditions. (J and K) Average GABAergic PSC amplitudes (J) and average failure rates (K) (chi-square test, n = 13–14 neurons, 4 mice, per condition). (L) Connection probability (chi-square test, n = 36–57 neurons, 5 mice, per condition). ^∗^p < 0.05 ^∗∗∗^p < 0.001. Bar plots show mean ± SEM. See also Figures S2, S3, and S5.

We next explored the role that network activity plays in the formation and plasticity of ChC circuits in the somatosensory cortex. Using designer receptors exclusively activated by designer drugs (DREADDs, specifically hM3Dq) (Urban and Roth, 2015) expressed in layer 2/3 pyramidal neurons in the somatosensory cortex, we increased network activity during the window of ChC synaptogenesis by delivering the DREADD agonist, clozapine-N-oxide (CNO), from P12 to P18 (Figure 2A). This manipulation resulted in an increase in activity (verified by cfos expression, Figure S2) in both pyramidal cells that expressed hM3Dq and neighboring cells that did not (referred to as hM3Dq-network), suggesting a network-wide increase in neuronal activity. Indeed, although increases in activity were initially confined exclusively to DREADD-expressing neurons (at P12; Figures S2A and S2B), activity spread to neighboring neurons in the network by the end of the CNO treatment (at P18; Figures S2C and S2D). We found that increased activity during this period resulted in a decrease in the overall number of pyramidal neurons contacted by a single ChC (Figure 2B), a finding that highlights the role of activity in molding the connectivity of inhibitory microcircuits. In parallel to this, we found that the number of axo-axonic synapses formed by single ChCs decreased significantly in both pyramidal neurons expressing DREADDs, as well as in neighboring DREADD-negative (hM3Dq-network) pyramidal cells (Figures 2C–2G), in agreement with a network-wide increase in activity (Figures S2C and S2D). Indeed, close inspection of the levels of cfos expression in the network also showed an increase in cfos-positive ChCs (Figures S2E and S2F), suggesting that increases in the activity of pyramidal cell networks lead to the recruitment of nearby ChCs. The connectivity between ChCs and pyramidal neurons was further studied functionally. GABAergic PSCs were recorded in pyramidal cells in response to optical stimulation of ChCs expressing channel-rhodopsin-2 (ChR2). Matching the morphological decrease in axo-axonic boutons, we found that the amplitude of evoked GABAergic PSCs also decreased, whereas the failure rates of synaptic transmission increased, in line with the idea that few synapses (∼5 boutons per ChC-pyramidal cell contact; Figures 2E–2G) contribute to the postsynaptic response (Figures 2H–2K). Finally, both morphological (Figure 2B) and functional (Figure 2l) measures of connectivity decreased significantly. Although the absolute values differed in each case, this discrepancy is likely explained by the unbiased low stringency rules used to measure morphological connectivity (see STAR Methods). We conclude that ChCs are sensitive to network activity during this early period of synapse formation, decreasing their output in a hyperactive environment.

Interneurons belong to a highly heterogeneous group of cells, often classified by their electrophysiological properties and the expression of specific genes (Kepecs and Fishell, 2014, Lim et al., 2018). Those that express parvalbumin (PV), for example, are typically fast spiking and comprise both basket cells and ChCs (Rudy et al., 2011, Tremblay et al., 2016). Importantly, both these PV interneuron types are thought to play an important role in controlling pyramidal cell output. However, despite their similarities, they show important differences, including their axonal morphology and the targeting of distinct subcellular compartments on pyramidal cells; whereas ChCs only target the AIS, basket cells predominantly target the soma and proximal dendritic domains (Rudy et al., 2011, Tremblay et al., 2016). We therefore also explored the effects of network activity on axo-somatic boutons to establish whether basket cells follow similar plasticity programs to ChCs. Contrary to what we observed for ChC axons, we detected an increase in the number of axo-somatic boutons onto hyperactive pyramidal cells, which likely belong to basket cells (Figures S3A–S3C). As expected from this morphological observation, the increase in axo-somatic synapses was also accompanied by an increase in spontaneous inhibitory postsynaptic current (sIPSC) frequency but not amplitude (Figures S3D–S3G) in hyperactive pyramidal cells, in agreement with previous findings (Kilman et al., 2002). In order to better understand the functional consequences of this form of developmental plasticity, we next set out to establish the properties of axo-axonic synapses at the AIS.

### Homeostatic Rules Drive Axo-axonic Synapse Plasticity

**Figure 3:**
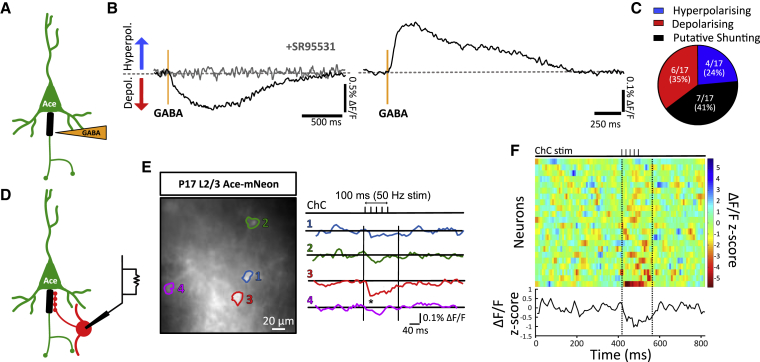
Axo-axonic Plasticity Matches GABAergic Polarity (A) Schematic showing iontophoresis experiment. A pipette containing GABA was placed near the AIS of a pyramidal neuron expressing Ace2N-mNeon. (B) Example traces obtained from imaging somatic responses to iontophoretic GABA application (orange area) at the AIS of two different cells. Left, a depolarizing response that is blocked by the application of 10 μM SR95531 (GABAzine). Right, example trace showing a hyperpolarizing response. (C) Classification of responses into hyperpolarizing (blue), depolarizing (red), and putative shunting (black) for all cells tested. (D) Schematic showing experimental logic: ChCs were patch-clamped to evoke APs, and responses were imaged from the soma of nearby pyramidal cells expressing Ace2N-mNeon. (E) Left, example image of Ace2N-mNeon cells, with somas outlined in different colors. Right, change in fluorescence of corresponding cells following ChC stimulation (top; 5 APs, 50 Hz) (^∗^denotes time-locked event 3x >baseline standard deviation in cell 3). Vertical lines denote start and end of expected response period, allowing up to 50 ms for decay of GABAergic responses (Figure 2I). (F) Heatmap showing *Z* scores of fluorescence responses following ChC stimulation for all 22 pyramidal cells tested. Mean *Z* score across all cells is shown below. See also Figures S4 and S6.

In the context of homeostatic forms of plasticity (Turrigiano and Nelson, 2004, Wefelmeyer et al., 2016) that rely on GABAergic inputs (Ma et al., 2019), the decrease of ChC axo-axonic synapses in hyperactive networks during development (Figures 2A–2G) is surprising. This discrepancy may be explained by recent work studying the switch in polarity of GABAergic synapses, as they transition from depolarizing to hyperpolarizing/shunting during development, along different subcellular compartments (Rinetti-Vargas et al., 2017). We therefore explored the polarity of axo-axonic synapses at the AIS during this early period of synaptogenesis by performing voltage imaging of pyramidal neurons in acute slices obtained at P14–P18. This approach provided a readout of changes in membrane potential in responses to GABA, delivered either by local iontophoresis or by ChC stimulation without perturbation of the intracellular milieu or of the resting membrane potential of the neuron. We first carried out local GABA iontophoresis at the AIS of pyramidal neurons expressing the genetically encoded voltage indicator Ace-mNeon (Gong et al., 2015) and grouped responses into three different categories: depolarizing, hyperpolarizing, and non-responsive (presumed shunting) (Figure 3A; see Figure S4 for voltage sensitivity). In agreement with previous findings (Rinetti-Vargas et al., 2017), GABA tended to produce mainly depolarizations at the AIS that were sensitive to GABA_A_ receptor antagonists during this developmental period (Figures 3B and 3C). Similar experiments applying GABA onto the soma of pyramidal neurons also resulted in an overall depolarization of the membrane, suggesting there are no obvious differences in GABA polarity between the soma and the AIS at this developmental stage in somatosensory cortex. To further explore the polarity of axo-axonic synapses, we performed whole-cell patch clamping of ChCs to directly stimulate a single ChC and image voltage responses in neighboring pyramidal cells that expressed Ace-mNeon (Figure 3D). Although GABAergic postsynaptic potential responses were small (Figure 3E; note that depolarizations produce a drop in fluorescence), we saw a clear overall depolarization below baseline during the stimulation period (5 action potentials at 50 Hz) that was apparent in individual cells, as well as in the mean response across all 22 cells (Figure 3F). Our results suggest that release of GABA by ChCs causes postsynaptic depolarizations in pyramidal neurons during the early period of synaptogenesis (P12–P18). These findings have important implications for interpreting the plasticity of axo-axonic synapses. We propose that the decrease in *depolarizing* axo-axonic synapses during chronic elevation of network activity is a homeostatic response that aims to stabilize circuit activity in the cortex.

### AIS Plasticity Accompanies the Plasticity of Axo-axonic Synapses

Previous work has demonstrated that the AIS of pyramidal cells is a plastic structure that can change in length (Baalman et al., 2013, Evans et al., 2015, Grubb et al., 2011, Kaphzan et al., 2011, Kuba et al., 2010) and position (Evans et al., 2013, Grubb and Burrone, 2010, Muir and Kittler, 2014, Wefelmeyer et al., 2015) in a homeostatic manner in response to chronic modulation of neuronal activity, using a variety of manipulations. We therefore investigated whether the structure of the AIS in pyramidal neurons was altered following increased activity. Measures of the length of the AIS showed that DREADD-expressing pyramidal cells, but surprisingly not neighboring hM3Dq-network cells, had shorter AISs (Figure S5A), which were matched by a decrease in intrinsic excitability (Figure S5B), a finding that is in line with homeostatic forms of plasticity previously observed at the AIS in other systems (Evans et al., 2015, Kuba et al., 2010). The confinement of AIS plasticity to DREADD-expressing neurons is puzzling and suggests that higher levels of activity are needed to drive this form of plasticity, which may only be achieved in those pyramidal cells directly activated by CNO. It also suggests that distinct mechanisms are likely used for AIS versus axo-axonic synapse plasticity (Wefelmeyer et al., 2015). Indeed, manipulations that drive the structural plasticity of the AIS have typically relied on chronic, strong changes in neuronal activity. In addition, it is also possible that the downstream intracellular cascades driven by the activation of hM3Dq, irrespective of neuronal activity, may also play a role here. The extent to which different mechanisms influence AIS plasticity *in vivo* will require further investigation. Altogether, our data show that the AIS and its axo-axonic synapses act as a plasticity hub that tightly controls the output of pyramidal neurons.

### Axo-axonic Synapse Plasticity Is Reversible

**Figure 4:**
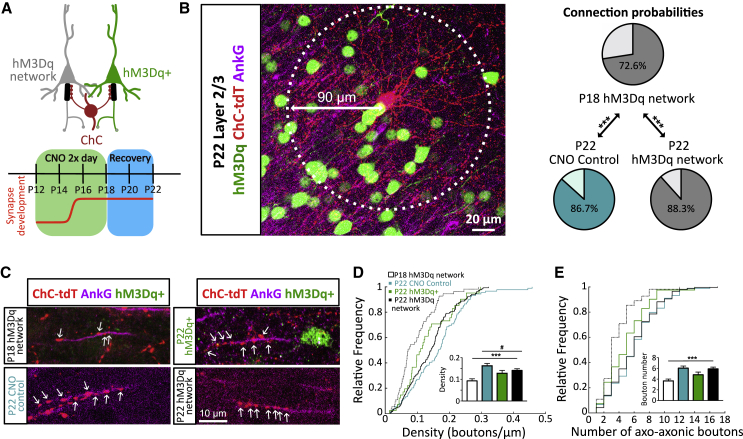
Axo-axonic Plasticity Is Reversible (A) Logic of experimental design and timeline of CNO application, including the recovery period. (B) Example image of a ChC and connection probabilities within a 90 μm radius at P22, after the recovery period (chi-square test, n = 180–390 AISs from 2–3 mice, per condition). (C) Example images of axo-axonic synapses and AISs following the recovery period. (D and E) Cumulative distribution of and average axo-axonic bouton density (D) and number (E) (#p < 0.05, Kruskal-Wallis test with Dunn’s post hoc comparisons; ^∗∗∗^p < 0.001 for P18 versus P22 hM3Dq-network, Mann-Whitney test. n = 41–89 neurons, from 2–3 mice, per condition). Bar plots show mean ± SEM. See also Figure S5.

Homeostatic forms of plasticity are typically reversible and follow the ongoing levels of neuronal or network activity (Turrigiano, 2011). We therefore investigated whether the plasticity of axo-axonic synapses and the AIS was permanent or whether it could be reversed if activity levels were normalized. Following 6 days of increased activity, mice were allowed to recover for a further 5 days without any CNO injections, after which axo-axonic synapse properties were assessed (Figure 4A). In agreement with a reversible form of plasticity, all measures of axo-axonic synapse connectivity were found to recover back to normal levels, indistinguishable from unstimulated neurons of the same age (Figures 4B–4E). Surprisingly, although the length of the AIS also recovered, it grew beyond control levels (Figures S5C and S5D), suggesting some kind of rebound effect during the recovery period. Together, our findings show that network activity reversibly controls the output of pyramidal cells during development by modulating both the structure of the AIS as well as the axo-axonic synapses that form onto it.

### Directly Activating ChCs Elicits Axo-axonic Synapse Plasticity

**Figure 5:**
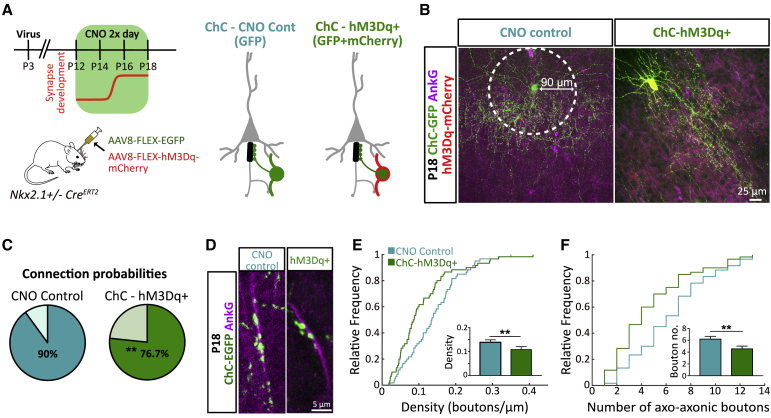
Axo-axonic Plasticity Is Cell Autonomous (A) Timeline of CNO delivery (top left) and strategy for viral delivery of GFP and hM3Dq to ChCs (bottom left). Experimental conditions of viral strategy (right). For CNO control, those ChCs infected with GFP virus only were analyzed, whereas, for the hM3Dq^+^ condition, ChCs expressing both GFP and hM3Dq-mCherry were analyzed. (B) Example images of P18 control and hM3Dq^+^ ChCs. (C) Connection probability within a 90 μm radius (white circle in B) for control and hM3Dq^+^ ChCs. chi-square test, n = 120–150 AISs per condition, from 3 mice. (D) Example images of P18 control and hM3Dq^+^ axo-axonic boutons. (E and F) Cumulative distribution of and average axo-axonic bouton density (E) and number (F). ^∗^p < 0.01 Mann-Whitney test, n = 60 AISs per condition, from 3 mice. Bar plots show mean ± SEM. See also Figure S5.

How do ChCs read out network activity to modify their output? Increases in network activity are likely to drive activity in ChCs through local intracortical connections (Lu et al., 2017), and indeed we found elevated levels of cfos expression in ChCs after increasing pyramidal cell activity (Figures S2E and S2F). To establish whether axo-axonic synapse plasticity requires the activity of pyramidal cells or can be driven by exclusively increasing the activity of ChCs in a cell-autonomous manner, we expressed DREADDs (hM3Dq) solely in ChCs (Figures 5A and 5B). Delivery of *hM3Dq* was achieved by postnatal (P2) injection of viral particles (AAV8-hSyn-DIO-hM3Dq) into layer 2/3 of somatosensory cortex in Nkx2.1-CreER^+/−^ mice. To obtain stable fluorescence for accurate morphological reconstructions we also expressed EGFP, also delivered virally (AAV8-CAG-DIO-EGFP). The low injected volume together with the sparsity of *Cre-*expressing ChCs resulted in a low infection efficiency that typically labeled a single hM3Dq-expressing ChC within layer 2/3 of somatosensory cortex. Control cells were taken from ChCs that expressed only EGFP and not hM3Dq, that were also found within the somatosensory cortex. Activation with CNO resulted in a similar reduction in synapse number and connection probability when compared to the plasticity driven by increases in pyramidal cell activity (Figures 5C–5F). This manipulation did not result in any significant changes in the size of the AIS of pyramidal neurons (Figures S5E and S5F), although there was a trend toward a decrease in AIS length (see rightward shift in cumulative distribution plot) that may reflect a homeostatic response to the activation of depolarizing axo-axonic synapses. Our results are consistent with the idea that ChCs sample the ongoing levels of activity in local pyramidal cell circuits (Figures S2E and S2F) affecting their own activity levels and altering their output accordingly.

### Axo-axonic Synapse Plasticity Switches Direction with Age

**Figure 6:**
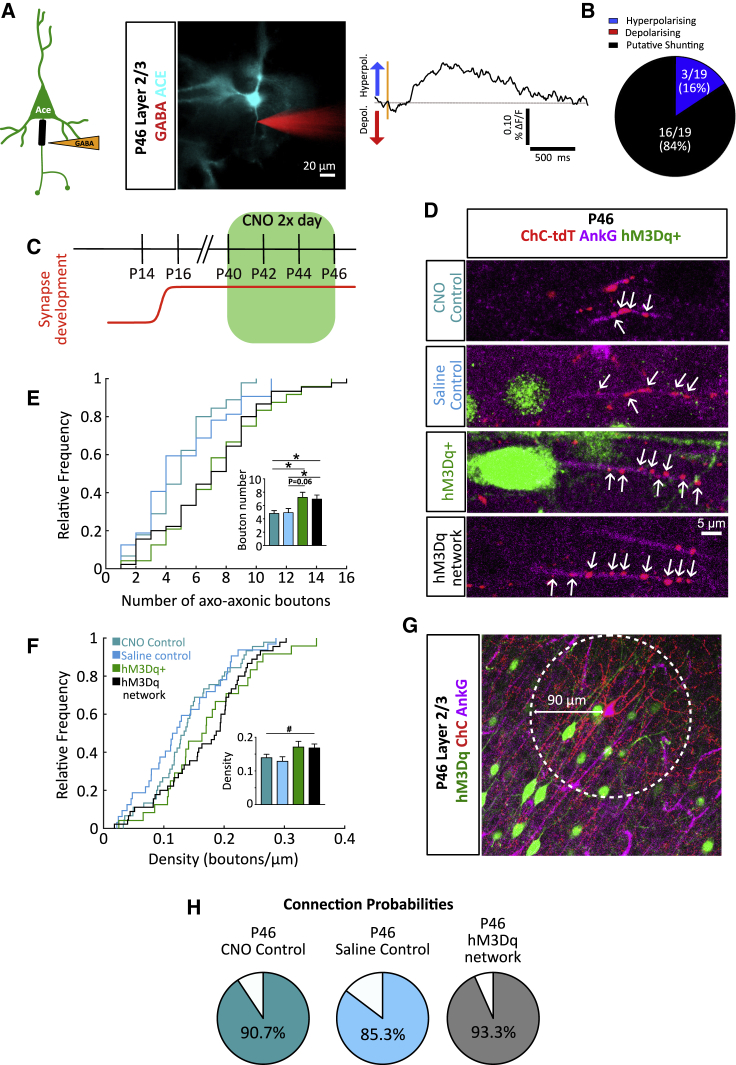
Axo-axonic Plasticity Matches Developmental Changes in GABAergic Polarity (A) Left, schematic and example image of iontophoresis experiment. A pipette containing GABA was placed near the soma (pipette in red) of a pyramidal neuron expressing Ace2N-mNeon (cyan). Right, example hyperpolarizing voltage response obtained from imaging somatic responses to iontophoretic GABA application (orange area) at the soma of a P46 pyramidal cell. (B) Classification of responses into hyperpolarizing (blue), depolarizing (red), and putative shunting (black) for all cells tested. (C) Timeline for CNO delivery in adult mice. (D) Example images of axo-axonic boutons in the different conditions at P46. (E and F) Cumulative distribution and average axo-axonic bouton number (E) and density (F) (# denotes p < 0.05 for one-way ANOVA without reaching significance in post hoc comparisons; ^∗^p < 0.05 denotes Kruskal-Wallis and Dunn’s post hoc comparisons test, n = 44–72 neurons per condition, 3–5 mice, per condition). (G and H) Example image (G) and connection probabilities (H) within a 90 μm radius (white circle) at P46 (chi-square test, n = 150–180 AISs, 3–4 mice, per condition). Bar plots show mean ± SEM. See also Figures S4–S6.

Previous studies have proposed that ChCs in adult brains are inhibitory, rather than excitatory (Lu et al., 2017, Rinetti-Vargas et al., 2017). We performed voltage imaging of pyramidal neurons expressing Ace-mNeon in acute cortical slices obtained from mice older than P40 (see Figures S4C and S4D for voltage sensitivity at P40). Contrary to the mainly depolarizing events observed in periadolescent slices (P12–P18), we found that local iontophoresis of GABA at the AIS caused either a hyperpolarizing or presumed shunting response, but no depolarizations (Figures 6A and 6B). A similar behavior was observed when GABA was applied to the soma (Figure S6). We therefore reasoned that, if ChCs follow homeostatic rules to control axo-axonic synapse number, the same increase in network activity performed in older animals should result in the opposite phenotype to that observed during development (P12–P18). Indeed, increasing the activity of cortical networks by delivery of CNO to DREADD (hM3Dq)-expressing pyramidal neurons from P40 to P46 resulted in a small increase in axo-axonic synapse number along the AIS (Figures 6C–6F). This effect was only observed as a change in the overall number of axo-axonic synapses (Figure 6E) but not synapse density (Figure 6F). Unlike manipulations during early development, the plasticity of axo-axonic synapses was not accompanied by clear changes in the size of the AIS (Figures S5G and S5H), nor in the number of postsynaptic pyramidal cells contacted by a single ChC (Figures 6G and 6H). The switch in the plasticity of ChC outputs can be explained as a homeostatic response that depends on the polarity of the synapse at the time. Whereas in mature brains axo-axonic synapses are inhibitory and therefore respond to hyperactivity by increasing their number, early in development, when they are depolarizing, they respond by decreasing their drive onto pyramidal cells.

## Discussion

GABAergic interneurons provide direct modulation of local circuit activity in the brain. However, the rules that govern the wiring of interneurons within local circuits remain unknown. Here, we focused on ChCs, a type of GABAergic interneuron that modulates circuit activity through axo-axonic synapses that form directly onto the AIS of pyramidal neurons. We find that ChCs extend axons to form synaptic contacts during a narrow temporal window and that this process is highly sensitive to network activity. More importantly, our study was able to probe the rules behind the plasticity of axo-axonic synapses, across the developmental switch in the polarity of GABAergic transmission. Whereas increasing network activity in developing brains (P12–P18), when axo-axonic synapses are depolarizing, caused a *reduction* in synapse number, the same manipulation in young adults (P40–P46), when axo-axonic synapses are inhibitory, resulted in a small *increase* in synapse number. Together, our data show that the plasticity of ChC axo-axonic synapses in the cortex is governed by homeostatic rules that control the number and strength of contacts onto pyramidal cells.

### The Emergence of Axo-axonic Synapses

Understanding the process of synapse formation and refinement is critical to establishing how circuits in the brain are built. Studies that have mapped the formation of excitatory synapses in the brain have uncovered important mechanisms that modulate these early events (Andreae and Burrone, 2014, Favuzzi and Rico, 2018). However, the emergence of GABAergic inputs that arise from the local axonal projections of interneurons is less well understood. Our study provides an *in vivo* description of the growth of a GABAergic interneuron as it forms synaptic contacts onto pyramidal neurons. We find that ChC axons undergo a dramatic increase in arbor size during a narrow developmental window in the third postnatal week, which coincides with an increase in axo-axonic synapse number at the AIS of pyramidal neurons. This burst in ChC wiring occurs relatively late when compared to the synaptogenesis of other interneurons, such as basket cells and somatostatin interneurons, which appear to form all their connections within the first 2 postnatal weeks (Favuzzi et al., 2019). Importantly, the period over which axo-axonic synapses emerge in the somatosensory cortex coincides with the start of active exploratory whisking that dramatically increases sensory driven activity in the network (Grant et al., 2009, Landers and Philip Zeigler, 2006). Although sensory deprivation experiments during this period show that activity plays an important role in the establishment of horizontal cortico-cortical connections (Fox, 1992), the interplay between network activity and GABAergic synapse formation remains elusive. In that regard, ChCs provide a particularly good model for tackling these unanswered questions.

### Plasticity of Axo-axonic Synapses

Since the early work describing the emergence of ocular dominance columns in the visual cortex (LeVay et al., 1980, Wiesel and Hubel, 1963), the role of activity in sculpting the process of circuit wiring has been an area of intense research. Although there is plenty of evidence to suggest that activity plays an important role in the later stages of circuit refinement (Huberman et al., 2008, Katz and Shatz, 1996, Sanes and Lichtman, 1999), more recent findings have also implicated activity in modulating the earlier process of synapse formation itself (Andreae and Burrone, 2014, Morgan et al., 2011, Soto et al., 2012). Specifically in interneurons, activity can play a role in surprisingly early events, including neuronal migration (De Marco García et al., 2011), developmental apoptosis (Denaxa et al., 2018, Priya et al., 2018, Wong et al., 2018), as well as dendritic and axonal morphology (De Marco García et al., 2011). We find that the wiring of axo-axonic synapses is highly sensitive to ongoing network activity and follows homeostatic plasticity rules, where the direction of synaptic plasticity acts in opposition to ongoing network activity levels. Similar homeostatic plasticity rules have been implicated in the process of synapse formation between specific neurons in the retina during development (Tien et al., 2017), suggesting that these wiring rules may be more widely used in the brain. From a network point of view, it appears that the overall goal of this wiring modality in ChCs is to stabilize local microcircuits in the cortex through changes in the connectivity and strength of axo-axonic synapses. Indeed, there is evidence to suggest that other GABAergic interneurons may also follow similar strategies (Kilman et al., 2002). For example, chronic modulation of individual pyramidal cell output in the visual cortex results in a homeostatic compensation of inputs from PV positive cells, with no effect on inputs from somatostatin cells (Xue et al., 2014). The idea of interneurons acting as local homeostats is appealing. At the single-cell level, they can act to stabilize neuronal activity by modifying the properties of different subcellular compartments, including dendrites, soma, and the AIS (Wefelmeyer et al., 2016), to avoid excessive levels of activity. From a network stance, the local connections formed by interneurons provide an ideal way of keeping microcircuits stable, preventing extreme levels of activity by modulating the strength of inhibition (Xue et al., 2014).

The fact that similar forms of plasticity were observed when ChCs were activated directly (by optogenetics) or indirectly (through activation of pyramidal cell networks) suggests that ChC activity is likely being driven by the local network. This is also supported by the increase in cfos-expressing ChCs observed following network stimulation. ChCs must therefore be receiving inputs from neighboring pyramidal neurons, probably within layer 2/3 of the cortex. Even though ChC dendrites extend mainly into layer 1, evidence for innervation of ChCs by local pyramidal neurons exists in the rodent prelimbic cortex (Lu et al., 2017), as well as the human cortex (Szabadics et al., 2006). Importantly, in the somatosensory cortex, *in vivo* recordings from ChCs showed that, although they rarely fire action potentials spontaneously and are only weakly driven by whisker stimulation, they respond vigorously (more than other interneurons) to overall increases in network activity driven by local application of bicuculine (Zhu et al., 2004). These findings place ChCs as a reliable sensor of network activity that can acutely modulate local cortical activity. Together with the network-wide homeostatic plasticity of axo-axonic synapses described here, ChCs appear to play a prominent role in both the acute and long-term stability of cortical networks.

### Plasticity across the GABA Polarity Switch

An important milestone in brain development is the switch in polarity of GABAergic transmission (Ben-Ari, 2002). Early in development, chloride levels in pyramidal neurons are high, such that activation of the chloride-conducting GABA receptors results in depolarizing responses that drive early forms of network activity (Bonifazi et al., 2009, Cossart, 2014). The expression of the chloride transporter KCC2 in pyramidal neurons during development causes a decrease in intracellular chloride that turns GABA into an inhibitory neurotransmitter, resulting in a switch in GABA polarity with age (Ben-Ari, 2002). Although this appears to hold true for most GABAergic synapses, the release of GABA from ChC axo-axonic synapses onto the AIS has been proposed to be excitatory even after the switch in GABA polarity. Lacking the transporter needed for lowering chloride levels, the AIS was proposed to be a subcellular compartment of high local chloride concentration, where GABA transmission can excite pyramidal neurons (Khirug et al., 2008, Szabadics et al., 2006, Woodruff et al., 2011), although these findings have been disputed (Glickfeld et al., 2009, Lu et al., 2017). The controversy over the polarity of axo-axonic synapses has been mostly reconciled by a recent study showing that the reversal potential of GABA at the AIS becomes hyperpolarized during development, but much later than in proximal dendritic compartments (Rinetti-Vargas et al., 2017). Indeed, GABA remains depolarizing at the AIS beyond the third postnatal week, a time period that spans the burst in synaptogenesis described above. One important drawback of previous experiments has been the reliance on relatively invasive techniques to measure the reversal potential of GABA. We have implemented recently developed genetically encoded voltage sensors (Gong et al., 2015) to measure postsynaptic potentials in a non-invasive manner that does not affect intracellular chloride levels or the resting membrane potential of the cell. Using this novel approach, we found that both local GABA iontophoresis at the AIS as well as activation of axo-axonic boutons can cause depolarizing responses in pyramidal cells during the window of synaptogenesis. However, GABA iontophoresis onto the AIS of more adult neurons (>P40) resulted in inhibitory responses, in agreement with previous findings (Rinetti-Vargas et al., 2017). A surprising, although tangential, finding is that GABA iontophoresis onto the soma followed a similar developmental trajectory to that observed at the AIS, with GABA remaining depolarizing at P14–P18. This result suggests that somatic GABAergic inputs may continue to be depolarizing beyond the usual period of early development in the somatosensory cortex, although whether they are ultimately excitatory or inhibitory at this age remains an open question.

More importantly, we were able to assess the plasticity of axo-axonic synapses during the period of depolarizing GABA, a feature previously unexplored in GABAergic interneurons. We found that the plasticity of axo-axonic synapses at the AIS is highly sensitive to network activity, such that increases in activity caused a decrease in depolarizing axo-axonic synapses. Surprisingly, the switch in GABA polarity, which converts axo-axonic contacts into inhibitory synapses, caused a corresponding change in the direction of the plasticity. In young adults, increases in network activity therefore resulted in a small, but measurable, increase in inhibitory axo-axonic synapses. It appears that these GABAergic synapses faithfully follow homeostatic forms of plasticity, regardless of their polarity. It is unclear, however, whether ChCs are able to sense the polarity of their output and switch the direction of synaptic plasticity accordingly. A more pragmatic explanation would argue that the parallel switch in polarity and synaptic plasticity simply relies on similar developmental trajectories that do not depend on sensing the polarity of postsynaptic responses. Regardless of the mechanism, the fact that these events are matched in time suggests ChCs are well placed to stabilize network activity in the cortex. More broadly, we need to understand how other interneurons that synapse onto different subcellular compartments behave in similar conditions. This knowledge would allow us to better understand how GABAergic circuits come together to modulate and stabilize network activity during development.

## STAR★Methods

### Key Resources Table

**Table:** 

REAGENT or RESOURCE	SOURCE	IDENTIFIER
**Antibodies**		

Anti-cFos-mouseϒ1	Santa Cruz Biotechnology	Cat# sc-166940; RRID: AB_10609634
Anti-cFos-guinea pig	Synaptic Systems	Synaptic Systems Cat# 226 005; RRID: AB_2800522
Anti-NeuN-rabbit	Millipore	Millipore Cat# MAB377; RRID: AB_2298772; clone 13E6
Anti-Mouse2b-Alexa Fluor 488	ThermoFisher	Thermo Fisher Scientific Cat# A-21141; RRID: AB_141626
Anti-Mouse2b-Alexa Fluor 647	ThermoFisher	Thermo Fisher Scientific Cat# A-21242; RRID: AB_2535811
Anti-Rabbit –Alexa Fluor 568	ThermoFisher	Thermo Fisher Scientific Cat# A-11011; RRID: AB_143157
Anti-Chicken-Alexa Fluor 488	ThermoFisher	Thermo Fisher Scientific Cat# A-11041; RRID: AB_2534098
Anti-mouseϒ3- Alexa Fluor 647	Jackson ImmunoResearch	RRID: AB_2338920
Anti-mouseϒ1- Alexa Fluor 647	Jackson ImmunoResearch	RRID: AB_2338916
Anti-tdTomato-Rabbit - Living Colors® DsRed Polyclonal Antibody	Takara Bio	Takara Bio Cat# 632496; RRID: AB_10013483
Anti-AnkyrinG-Mouse2b	Neuromab	RRID: AB_10673449; NeuroMab clone N106/65
Anti-GFP-chicken	Abcam	Abcam Cat# ab13970; RRID: AB_300798
Anti-VGAT-mouseϒ3	Synaptic Systems	Cat. No. 131 011; Clone117G4

**Bacterial and Virus Strains**		

AAV8-hSyn DIOhM3D(Gq)mCherry	UNC - Vector Core	N/A
AAV8-CAG-FLEX- GFP	UNC - Vector Core	N/A

**Chemicals, Peptides, and Recombinant Proteins**		

NBQX	Santa Cruz Biotechnology	sc-222048; CAS: 479347-86-9
SR 95531	Cambridge Bioscience	CAY14585; CAS: 104104-50-9
GABA	Tocris	Cat. No. 0344
CGP55845	Tocris	Cat. No. 1248
Tamoxifen	Sigma-Aldrich	Cat 85256-50MG
Clozapine-N-Oxidase	Enzo Life Sciences	BML-NS105-0005
AP-5	Cambridge Bioscience	CAY14539; CAS: 79055-68-8
Fluoromount-G®	Southern Biotech	Cat. No.0100-01
Vectashield-DAPI-G®	Vector Laboratories	Cat. No. H-1200

**Experimental Models: Organisms/Strains**		

Nkx2.1-CreER - *Nkx2-1*^*tm1.1(cre/ERT2)Zjh*^/J	The Jackson Laboratory (JAX)	IMSR Cat# JAX:014552; RRID: IMSR_JAX:014552
Ai9 (tdTomato) - B6.Cg-*Gt(ROSA)26Sor*^*tm9(CAG-tdTomato)Hze*^/J	The Jackson Laboratory (JAX)	IMSR Cat# JAX:007909; RRID: IMSR_JAX:007909
Ai32(Chr2) - B6;129S-Gt(ROSA)26Sor^tm32(CAG-COP4∗H134R/EYFP)Hze^/J *Mus musculus*	The Jackson Laboratory (JAX)	IMSR Cat# JAX:012569; RRID: IMSR_JAX:012569

**Recombinant DNA**		

Lv-CamkII-Ace2N-4AA-mNeon	Ace2N-4AA-mNeon distributed by Biolife Valley (Gong et al., 2015, *Science*)	S-ACE-P-001
pCAGGS-hM3D(Gq)-IRES-GFP	G Neves (Denaxa et al., 2018)	N/A
pCAGGS-hM3D(Gq)-IRES-RFP	G Neves (Denaxa et al., 2018)	N/A

**Software and Algorithms**		

MATLAB 2016-2019	MathWorks	https://www.mathworks.com/products/matlab.html
Simple Neurite Tracer	Fiji (ImageJ)	https://imagej.net/Simple_Neurite_Tracer
AIS fluorescence analyzer (used for analysis of AIS plasticity only)	Custom MATLAB code from	https://github.com/alejandropan/pan-vazquez-wefelmeyer-et-al
Noise removal for voltage imaging	Custom MATLAB code	https://github.com/alejandropan/pan-vazquez-wefelmeyer-et-al
Prism7	GraphPad	https://www.graphpad.com/scientific-software/prism/
Neuromatic package – Igor Pro	WaveMetrics	http://www.neuromatic.thinkrandom.com/

### Lead Contact and Materials Availability

Further information and requests for resources and reagents should be directed to and will be fulfilled by the Lead Contact, Juan Burrone (juan.burrone@kcl.ac.uk).

This study did not generate new unique reagents.

### Experimental Model and Subject Details

#### Animals

Male and female mice were used for all experiments and randomly assigned to experimental groups. Mice were housed in individually ventilated cages and provided with *ad libitum* food and water. Pups were kept with their mother until weaning age P21. Delivery of viral vectors was done in the Nkx2.1-CreER^+/−^ line (JAX014552, see Key Resources Table). For morphological analysis and voltage imaging, Nkx2.1-CreER^+/−^ mice were crossed with the Ai9 (JAX007909, see Key Resources Table) line. For measuring the intrinsic excitability of chandelier cells, Nkx2.1-CreER^+/−^ mice were crossed with both the Ai9 and the Ai32 line (JAX012569, see Key Resources Table). For measuring the electrophysiological properties of the axo-axonic synapses Nkx2.1-CreER^+/−^;Ai32 mice were used. For measuring spontaneous GABAergic PSC frequency, Nkx2.1-CreER^+/−^;Ai32 were used. Finally, for recording the intrinsic excitability of pyramidal cells after DREADDs treatment and iontophoresis experiments, Ai9 mice were used. Both homozygous and heterozygous mice were used. All procedures were carried out in accordance with the UK Animal Scientific Procedures Act.

#### Tamoxifen induction

For the optimal labeling of ChCs, Tamoxifen dependent Cre recombination was induced at P2. For experiments using *Cre*-dependent viral vectors, Tamoxifen was injected at P3, on the day of viral injection. Tamoxifen (Sigma Aldrich) was dissolved in corn oil (6 mg/mL) and injected intraperitoneally at a dose of 100 μg/g.

### Method Details

#### Cranial windows

Cranial windows were implanted covering somatosensory cortex as in previous studies (Wong et al., 2018). P11-P12 mice were anaesthetised with 2% isoflurane in a nose-clamp and body temperature maintained at 37°C with a heating pad. The local anesthetic Bupivacaine was injected sub-cutaneously (10 μl of 0.5 μg/ml) and the anti-inflammatory dexamethasone was applied intramuscularly (10 μl of 38 μg/ml solution) at the start of the procedure. The scalp was cleaned with betadine and cut open to expose the cranium. Lidocaine Hydrochloride (1% w/v) was then applied briefly to the cranial surface before the periosteal tissue was carefully removed with a scalpel. The cranial surface was then cleaned with Ringer’s solution and a few drops of betadine. A custom-made metal head-post (design by Carl Petersen lab) was glued to the right hemisphere with cyanoacrylate glue (Henkel). Dental cement (Paladur) was applied around the head-post and the cranium to reinforce the implant. A 3 mm craniotomy was opened over the somatosensory cortex with a dental drill until the dura was exposed. Care was taken not to cross the bone sutures. Cortex buffer (NaCl [123.35 mM], KCl [5 mM], Glucose [10 mM], HEPES [10 mM], CaCl2 [2 mM], MgSO4 [2 mM]) was applied to the exposed surface and a 3 mm glass coverslip (Harvard Apparatus) placed directly over the craniotomy, attached at the edges with cyanoacrylate glue and strengthened with dental cement. At the end of the procedure, mice were injected with saline and left to recover for 4 h - 1 day before imaging.

#### 2-photon *in vivo* imaging

Mice were anaesthetised with Ketamine/Xylazine. Imaging sessions lasted 2-3 hours. The same field of view was imaged over consecutive days. tdTomato was excited with a Ti-Sapphire laser (Coherent Chameleon) at 930 nm wavelength. Emitted light was collected by a GaAsP detector through a 1.0 NA, 20 × objective (Olympus). The excitation power was between 40 and 50 mW.

#### Histology

Mice were anaesthetised with an overdose of sodium pentobarbital and transcardially perfused with 10 mL of ice-cold saline solution followed by 20 mL of 1% (w/v) PFA in HEPES (pH 7.3). The brains were carefully removed and put in 1% (w/v) PFA solution overnight at 4°C. Brains were embedded in blocks of 6% agarose (Sigma) and sectioned coronally (100 μm thick) with a vibratome (Leica). Slices were kept in PBS supplemented with 0.05% Sodium Azide to improve preservation. On the day of staining, slices were incubated at RT for 2h in PBS-Triton (0.25%) supplemented with 10% Goat Serum (GS) (Sigma). This was followed by an overnight incubation in a primary antibody solution in PBS-Triton (0.25%)-GS (10%). On the following morning, slices underwent three 20 minute PBS-Triton (0.25%) washes, followed by a 2-hour incubation at room temperature in secondary antibody solution in PBS-Triton (0.25%)-GS (10%). The secondary antibody solution was washed off as before and slices were mounted with Fluoromount (Southern Biotech) mounting media, (samples used for cfos but not AIS analysis were mounted with DAPI-Vectashield (VectorLabs)). Mounted samples were kept at 4°C. The following primary antibodies were used: Anti-AnkyrinG-mouse-IGg2b (NeuroMab), Anti-DsRed-rabbit (Takara), Anti-GFP-chicken (Abcam), Anti-VGAT-mouse-IgG3 (Synaptic Systems), Anti-cfos-mouse-IgG1 (Santa Cruz), Anti-cfos-guinea pig (Synaptic Systems) (used for cfos staining of ChCs), Anti-NeuN-mouse-IgG1 (Millipore). Alexa Fluor conjugated antibodies (Invitrogen) were used as secondary antibodies.

#### In utero electroporation

CAG:hM3Dq:IRES:GFP, CAG:hM3Dq:IRES:RFP or CamKII-ACE2N-mNeon-4AA were expressed in pyramidal cells via in utero electroporation. In utero electroporation was performed in E15.5 pregnant mice. Females were anaesthetised with isoflurane 2.5% before the abdomen was opened and the uterine horns exposed. DNA was delivered to the embryos via injection into the lateral ventricles with a glass micropipette. A total of 0.5-1 μL of DNA solution was injected into each embryo. The DNA solution contained Fast Green (0.3% (w/v)) and DNA in TE buffer (1500-2000 ng/ml). Five square electric pulses (30 V, 50 ms) were passed at 1 s intervals using a squarewave electroporator (CUY21EDIT; NEPA GENE). All successfully electroporated mice were included in all analyses.

#### Expression of viral constructs in ChCs

AAV8-CAG-FLEX-GFP (UPenn Vector core) and AAV8-hsyn-hM3D(Gq)-mCherry (UNC Vector Core)(Krashes et al., 2011) were injected into L2/3 somatosensory cortex of P3 Nkx2.1-CreER^+/−^ mice. Mice were anaesthetised with 2.5% isoflurane. The following coordinates were used: Anterior-Posterior-axis: 2.7 mm, Dorso-Ventral: −0.3 mm, Lateral: −2.3 mm from Bregma. A total volume of 250 nL was infused at a flow rate of 50 nl/min. All injections were performed with a Nanoliter injector (WPI) controlled with a SMARTouch controller (WPI) as in Favuzzi et al. (2017) and Hinojosa et al. (2018). This resulted in very sparse labeling of ChCs, with typically only a single ChC expressing both hM3Dq and EGFP within layer 2/3 of somatosensory cortex. ChCs found within the somatosensory cortex that expressed only EGFP and not hM3Dq were used as control.

#### Clozapine-N-Oxide (CNO) treament

For DREADDS experiments, CNO (Enzo Life Sciences) was dissolved in 0.5% dimethyl sulfoxide (DMSO) and 0.9% saline to a final concentration of 0.5 mM. Once in solution, CNO was aliquoted and stored at −20°C until the day of use. Animals were injected intraperitoneally twice daily with saline (0.9%) with DMSO (0.5%) or CNO in DMSO (0.5%) at 1 mg/kg. The saline volume injected was calculated based on the CNO equivalent for the given weight of the animal. On the final day of the experiment animals were only injected once, 2h before perfusion.

#### Image acquisition of histology

A Nikon A1R inverted confocal microscope with a 40x water immersion objective (NA 1.1) was used for the acquisition of images from immunostained slices at 1024 × 1024 pixels resolution. For synaptic measurements, a 3x zoom was used. Stacks had a z-step of 1 μm. All images were acquired with NIS Elements software (Nikon).

#### Acute slice preparation for electrophysiological and optical recordings

Mice were anaesthetised with Isoflurane and their brains rapidly removed in cold dissection media (Sucrose 240 mM, KCl 5mM, Na_2_HPO_4_ 1.25 mM, MgSO_4_ 2 mM, CaCl_2_ 1 mM, NaHCO_3_ 26 mM and D-glucose 10 mM). 300 μm thick coronal slices were made with a V1000S vibratome (Leica) in constantly carbonated dissection media (95% O_2_, 5% CO_2_). Slices were transferred to an immersion storage chamber containing artificial cerebrospinal fluid (ACSF) with the following composition: NaCl (124 mM), KCl (5 mM), Na_2_HPO_4_ (1.25 mM), MgSO_4_ (2 mM), CaCl_2_ (2 mM), NaHCO_3_ (26 mM), D-glucose (20 mM). Slices were left to recover for 1 hour at room temperature before recording. For recording in adult neurons (P40+), brains were cut in cold NMDG-HEPES solution (NMDG 93 mM, HCl 93 mM, Glucose 25 mM, KCl 2.5 mM, NaH_2_PO_4_ 1.25 mM, HEPES 20 mM, NaHCO_3_ 30 mM, Thiourea 2 mM, Na-ascorbate 5 mM, Na-pyruvate 3 mM, CaCl_2_-2H_2_O 0.5 mM, and MgSO_4_-7H_2_O 10 mM). Additionally, after cutting, all slices were incubated for 10 min in pre-warmed 34°C NMDG-HEPES solution. This was followed by a 1h incubation in HEPES-ACSF holding solution (NaCl 92 mM, KCl 2.5 mM, NaH_2_PO_4_ 1.25 mM, NaHCO_3_ 30 mM, HEPES 20 mM, Glucose 25mM, Thiourea 2 mM, Na-ascorbate 5 mM, Na-pyruvate 3 mM, CaCl_2_·2H_2_O 2 mM, and MgSO_4_·7H_2_O 2mM).

#### Electrophysiology

Targeted whole-cell patch clamp recordings were made at the soma of ChCs or pyramidal cells in layer 2/3 under voltage and current clamp configurations. Acute slices were transferred to a recording chamber equipped with a BX51 (Olympus) microscope with Dodt gradient contrast optics. For targeted patching, tdTomato+ ChCs were illuminated with a 565 nm LED (2-pE system, CoolLED) which resolved the soma and axonal arbour of the cell. Slices in the recording chamber were constantly superfused with ACSF and carbonated (95% O_2_, 5% CO_2_). For all recordings, the AMPA receptor blocker NBQX (10 μM), and the NMDA receptor blocker APV (25 μM) were present in the bath. Patching electrodes were made from thick-walled borosilicate glass (O.D: 1.5mm, I.D: 0.86 um, Sutter Instruments) and polished with a Microforge MF-900 (Narishige) obtaining a resistance between 3-4 MΩ. Recordings were obtained with a Multiclamp 700B amplifier (Molecular Devices) and digitised with the Digidata 1440A digitizer (Molecular Devices). Data was acquired with the software Clampex (Molecular Devices) and Axon Multiclamp Commander Software (Molecular Devices). Signals were filtered at 10 kHz and sampled at 50 kHz. Pipette offsets were nulled before seal formation and pipette capacitance was compensated in the cell-attached configuration once a giga-seal was obtained. All measurements in voltage-clamp were made after series resistance compensation for 7 MΩ. Recordings were excluded if access resistance exceeded 30 MΩ. All recordings were done at room temperature. Membrane resting potentials were checked in *I* = 0 mode and passive features were recorded on-line. For intrinsic excitability measurements, 10 ms and 500 ms current injections of increasing amplitude (10 pA steps) were injected in current clamp configuration. For optogenetic stimulation of ChCs, acute slices were examined via a 2-photon microscope integrated into our patch clamp setup to confirm that there was no overlap and enough separation between the arbours of different ChCs in each slice. To measure GABAergic PSCs, pyramidal cells were held in voltage-clamp at −70 mV while ChCs were stimulated with light with 2 ms pulses and 20% LED intensity (470 nm). A 10 s interval was left between trials to allow for the full replenishment of the vesicular pool. For measuring spontaneous GABAergic PSCs, cells were held at −70 mV and recorded for 3 min.

#### Internal solutions for patch-clamp recordings

For intrinsic excitability measurements of chandelier and pyramidal cells the internal solution in the recording pipette contained: K-gluconate (130 mM), KCl (15 mM), 10 Na_2_Phosphocreatine (10 mM), HEPES (10 mM), MgATP (4 mM), GTP (0.3 mM) and EGTA (0.3 mM), pH was adjusted to 7.3 with KOH and to 320 mOsm. For recording GABAergic PSCs, a high chloride intracellular solution was used for data in Figures 1 and 2, containing KCl (150 mM), MgCl_2_ (4.6 mM), CaCl_2_ (0.1 mM), HEPES (10 mM), NaATP (4mM), NaGTP (0.4 mM) and EGTA (1 mM), pH was adjusted to 7.3 with KOH and to 320 mOsm. Because of the prevalent presence of action currents in > P18 and older animals, QX-314 (Tocris, 5 mM) was included in the solution for GABAergic PSC recordings in the experiments in Figure 2.

#### Voltage imaging

Acute slices were prepared from mice electroporated with the voltage indicator Ace2N-mNeon-4AA in prefrontal and somatosensory cortex. Slices were transferred to a recording chamber equipped with a BX51 (Olympus) microscope with Dodt gradient contrast optics, 470 nm & 565 nm LEDs (CoolLED) and an Evolve 512 EMCCD camera (Photometrics). The slices were continuously superfused with carbonated ACSF containing NBQX (10 μM, Tocris), and APV (25 μM, Tocris). In the iontophoresis experiments the GABA_B_R inhibitor CGP55845 (10 μM, Tocris) was also always present in the bath. Cells were illuminated with a 470 nm LED at 29 mW/mm^2^ (illumination intensity measured at the specimen plane), while imaging at *circa* 120 Hz with an EMCCD camera through a 40x, 0.8 NA water-immersion objective (Olympus). To image at this acquisition rate, 4x4 binning was used, giving a final resolution of 128x128 pixels. Images were acquired using μmanager software (Micro-manager, Vale Lab). Each recording consists of 40-50 repeats with the same stimulus. To obtain a baseline of camera noise, acquisition started 50 ms prior to LED illumination and finished 100 ms after. LED illumination, camera acquisition and stimulation were synchronized with a Digidata 1440A digitizer (Molecular Devices) through the software Clampex (Molecular Devices).

#### GABA Iontophoresis

After selecting an ACE-mNeon cell, an iontophoresis pipette was placed juxtaposed to the cell’s AIS or soma. Iontophoresis pipettes were made from thick-walled borosilicate glass (O.D: 1.5mm, I.D: 0.86 um, Sutter Instruments) obtaining a resistance of 60-100 MΩ. The pipettes were filled with a GABA solution containing: GABA (200 mM, pH 5.0), Alexa Fluor 568 (10 μM) and CGP55845 (10 μM, Tocris). Alexa 568 was used to visualize GABA application. CGP55845 was also included in the iontophoretic solution to assure GABA_B_R inhibition and prevent potential displacement of the blocker when GABA was ejected. A retention current of −30 nA was used when the pipette was in the bath to avoid spillage. A single pulse of 20 ms and 150 nA was used to eject the GABA solution from the pipette.

### Quantification and Statistical Analysis

#### Morphological analysis of AIS and axo-axonic boutons

For all morphological analyses, care was taken to include an equal number of AISs or cartridges from each animal. AISs and boutons were traced in 3D using the ROI manager and Simple Neurite Tracer (Fiji-ImageJ, see Key Resources Table for details). The fluorescent signal for the AIS fades on its ends and has its brightest points at the center. Therefore, to normalize AIS length when examining AIS plasticity, custom MATLAB code was used (Wefelmeyer et al., 2015, see Key Resources table for code availability). The algorithm assigns the points delimiting the region where intensity dropped to 20% of maximum fluorescence as the start and end points. All analyses were performed under blinded conditions. Due to the sparsity of chandelier cells in somatosensory cortex, individual cartridges could be related to specific chandelier cells. All chandelier cell varicosities overlapping an AIS were included in the analysis of bouton number and density. To establish a measurement independent of AIS structure, we also measured cartridge size. A cartridge was defined as a collection of 2 boutons or more that project in the same axes as the AIS they synapse onto. This measurement was based purely on axo-axonic cartridge morphology without taking into consideration whether the boutons in the cartridge were contacting an axon. For the analysis of contacts made by an individual chandelier, we cropped a 90 μm radius x 100 μm height cylinder around the soma of a chandelier cell as this is the area in which the majority of ChCs made their contacts (Inan et al., 2013), and blindly sampled 30 AIS. A contact was defined as the presence of at least one ChC varicosity overlapping an AIS. This morphological estimate of connectivity is higher than our electrophysiological estimate, presumably due to this low-stringency approach of considering any morphological overlap a contact. On the other hand, our electrophysiological approach is likely underestimating connectivity due to the combination of a small number of synaptic contacts with a low release probability. It is likely that a combination of the two (overestimation of morphological connectivity and underestimation of functional connectivity) explains the absolute difference in connection probability measured in each case.

#### Morphological analysis of basket cell boutons

The number of synapses at an individual soma was analyzed in a single plane as in Del Pino et al. (2013). Somas were stained with NeuN and GABAergic synapses with VGAT. Thresholding for VGAT and NeuN immunostaining was adjusted in each individual image to ensure optimal segmentation. Segmentation and synapse counting was done automatically. Synapses were segmented with a watershed algorithm (Fiji-ImageJ). Synapses were included if they were within a 1 μm radius of the segmented soma. Density of synapses was calculated by dividing synapse number over the perimeter of the soma. All analysis was done under blind conditions.

#### Analysis of cfos immunofluorescence

To analyze the effect of DREADDs and its ligand on activity, mice were injected with CNO or saline 2h before perfusion and stained for cfos, a gene with activity dependent expression. A fluorescence threshold of 1.2x background fluorescence was set to classify pyramidal cells as cfos+ and a threshold of 2 standard deviations above background fluorescence was set to classify ChCs as cfos+. For measuring the effects of CNO on hM3Dq+ cells in hM3Dq-networks, cells were blindly selected before measuring their cfos immunofluorescence profile. All analyses were done in Fiji – ImageJ.

#### Analysis of electrophysiological measurements

Input-output curves were generated with custom MATLAB scripts. Two types were used: 1) output in action potential (AP) frequency as a function of the current injected (pA) 2) output in AP frequency as function of current density (pA/Pf). This is the same as 1) but normalized for the capacitance of the cell, which correlates with its size. For the measurements of the voltage threshold, voltage threshold was determined by time of the first peak of the jerk (third derivative of voltage with respect to time). For the measurement of GABAergic PSC amplitudes, all analysis was done with the IGOR (Wavemetrics) package Neuromatic (Rothman and Silver, 2018). GABAergic PSCs were averaged over 15 sweeps and the maximum amplitude recorded. For the measurement of spontaneous GABAergic PSCs, template matching to a second exponential curve was used to automatically record the number of events. The parameters for template matching were as follows: rise time, 1 ms, decay time, 20 ms, zero-baseline before waveform, 1 ms, total template waveform time, 40 ms. For the measurement of release failures, only cells with at least two successfully evoked postsynaptic potentials were included. Failures were defined as the absence of postsynaptic potentials in response to light stimulation on the presynaptic ChC.

#### Analysis of voltage imaging recordings

For analyzing voltage imaging recordings from evoked ChC activity, an ROI was selected covering the soma of the pyramidal cell of interest. From this ROI, the fluorescence intensity profile over time for each repeat (stimulation protocol repeat) was obtained. Camera noise was estimated as the average over a 10 frame window with no LED illumination and was subtracted from the profile. The resulting traces were parsed through a custom-made bleach-correction algorithm (MATLAB). In this algorithm, the intensity profile for each repeat was fitted to a double exponential curve excluding the points corresponding to expected evoked response time. From the resulting trace ΔF/F was calculated. F was determined as the average bleach-corrected baseline fluorescence of the last 10 frames prior to the evoked event. The resulting ΔF/F traces for each repeat were then averaged. Finally, the averaged trace was smoothed by applying a 3-frame moving average filter. For analyzing voltage imaging recordings from the iontophoresis experiments, a slight variation to this algorithm was introduced to enable bleach correction for traces with long responses and little baseline for fitting. In these experiments the bleach correction algorithm was trained in non-stimulation repeats in the same cells, where no GABA was released.

#### Statistics

Statistical analysis was performed with Prism 6 (GraphPad) and MATLAB. Before hypothesis testing, D’Agostino normality test was used in all datasets to determine whether the data followed a normal distribution. Depending on the outcome, the parametric One-Way-ANOVA or non-parametric Kruskal-Wallis test was used when comparing over more than 2 groups. This was followed by a multiple comparison post hoc analysis with a multiple comparisons correction. For comparison between two groups, a Student’s t test or the non-parametric Mann-Whitney test was used, depending on normality. Correlations were tested using Pearson’s or Spearman’s method depending on normality. For comparisons of frequency, a X^2^ test was used. Values are presented as mean ± s.e.m. Statistical significance was established at p < 0.05. Statistical details can be found on the figure legends.

### Data and Code Availability

Original data and analysis programs will be provided by the corresponding author (J.B.) upon reasonable request. The custom MATLAB code for measuring AIS length and fitting voltage responses is available in Github (See Key Resources Table for details).

## Supplemental Information

Document S1. Figures S1–S6

Document S2. Article plus Supplemental Information
