# Supplementary material for: Activity-Dependent Plasticity of Axo-axonic Synapses at the Axon Initial Segment
Source: Neuron. 2020 Apr 22;106(2):265–276.e6. doi: 10.1016/j.neuron.2020.01.037 (PMC7181187; doi:10.1016/j.neuron.2020.01.037)
Supplement: Document S1. Figures S1–S6 [file mmc1.pdf]

Neuron, Volume *106*

Supplemental Information

Activity-Dependent Plasticity of Axo-axonic

Synapses at the Axon Initial Segment

Alejandro Pan-Vazquez, Winnie Wefelmeyer, Victoria Gonzalez Sabater, Guilherme Neves, and Juan Burrone

Supplementary Items

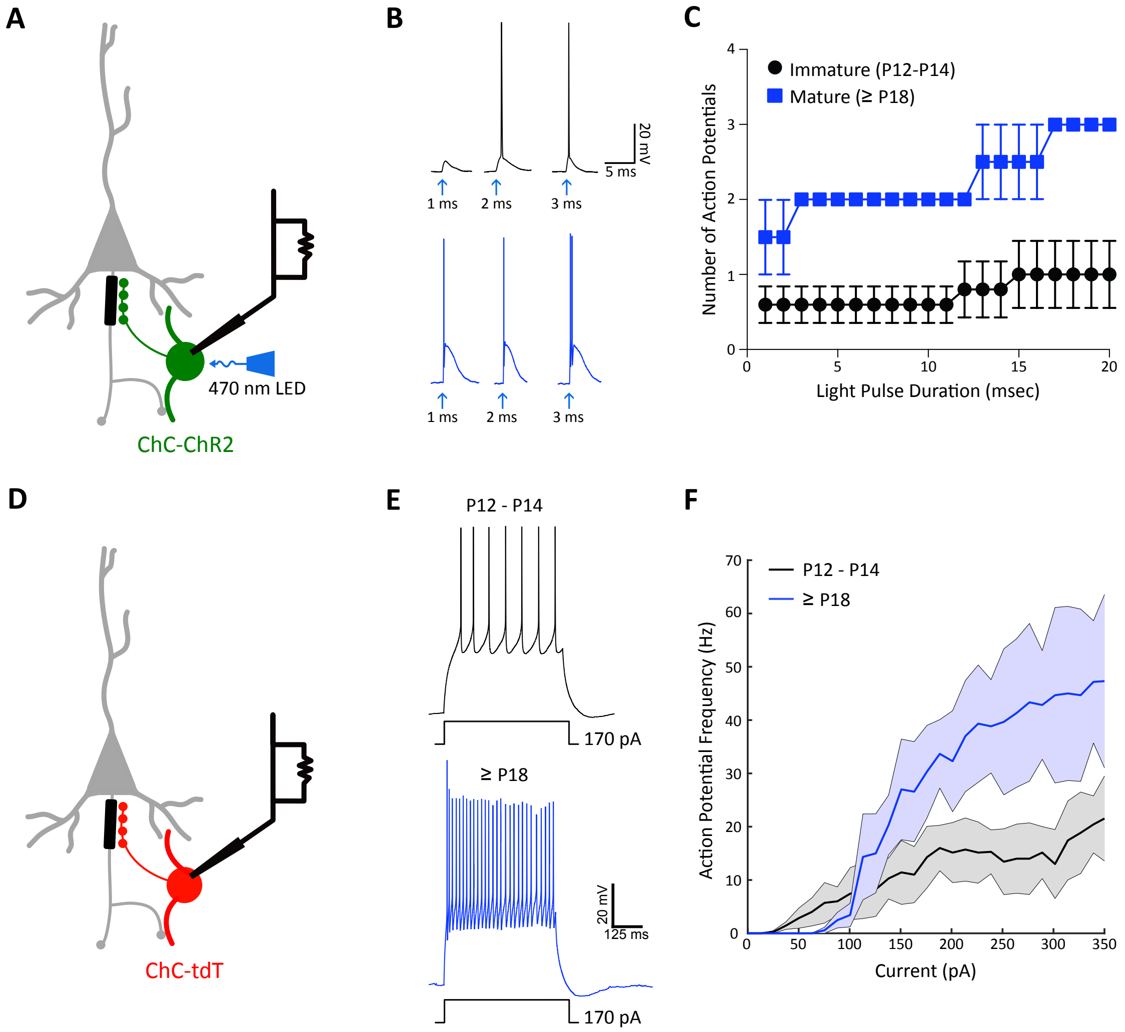

Figure S1. ***Related to Figure 1.*** **Optogenetic stimulation and excitability of ChCs throughout development. (a)** Chandelier cells were patched in whole-cell configuration from Nkx2.1-CreER$^{+/-}$;Ai32 mice and stimulated with a 470 nm LED. **(b)** Representative action potentials generated with 1, 2 and 3 ms light pulses (arrows) in immature (black) and mature (blue) ChCs. **(c)** Input-output curves showing action potential number as a function of light pulse length in immature (black) and mature (blue) ChCs. Values shown are mean ± s.e.m. Immature: n = 5 ChCs, n = 3 mice; Mature: n = 2 ChCs, n = 2 mice. **(d)** Chandelier cells were patched in whole-cell configuration during development. **(e)** Representative traces from 500 ms current injections in immature (P12-P14, black) and mature (≥P18, blue) ChCs. **(f)** Input-output curves showing action potential frequency in response to the current injected. Centre line for each group represents mean firing rate, shadows represent s.e.m. Immature: n = 7 ChCs, n = 3 mice; Mature: n = 6 ChCs, n = 4 mice

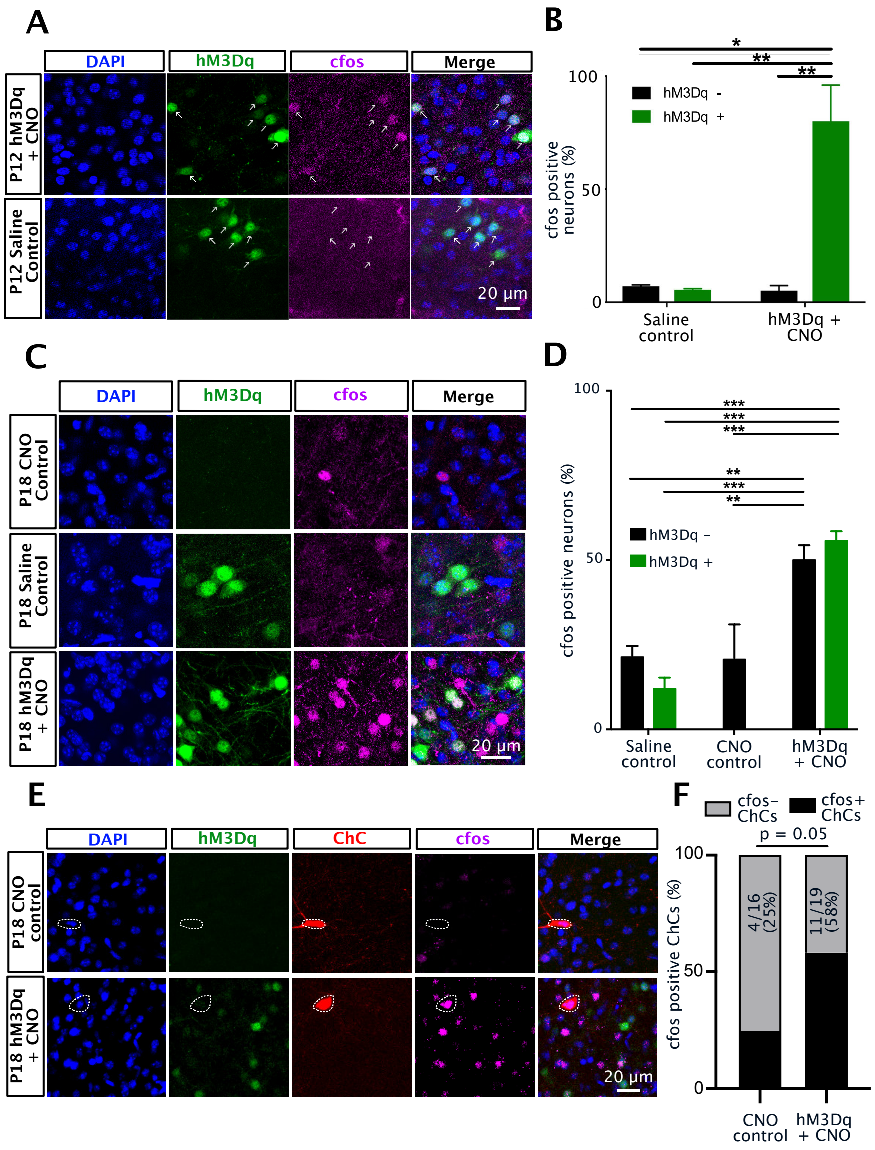

Figure S2. ***Related to Figure 2.*** **Cfos expression after CNO injection in hM3Dq+ and surrounding pyramidal cells. (a)** Representative images of cfos expression in L2/3 cells in somatosensory cortex following a CNO or saline injection in P12 mice. **(b)** Percentage of cfos positive cells in saline and CNO injected animals (Chi-Square test, N = 300 cells, 3 mice, per condition). **(c)** Representative images of cfos expression in L2/3 cells following repeated injections of CNO/saline from P12-P18. **(d)** Percentage of cfos positive cells across conditions (Chi-Square test, N = 300 cells, 3 mice, per condition). **(e)** Representative images of cfos expression in ChCs in hM3dq (+) networks and CNO control. **(f)** Percentage of cfos positive ChCs across conditions (Chi-Square test, N = 35 cells, 2 mice). *p<0.05, **p<0.01, ***p<0.001, bar plots show mean ± s.e.m.

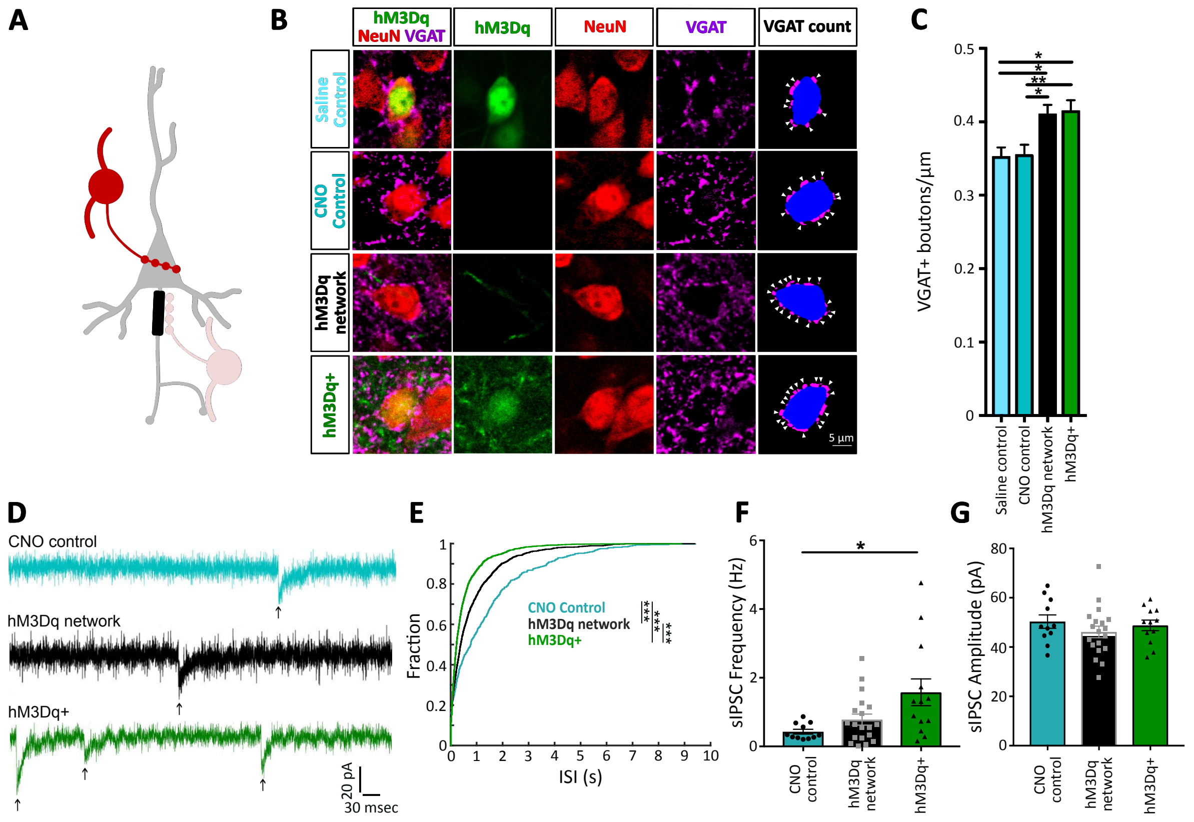

Figure S3. ***Related to Figure 2.*** **High network activity decreases the number of GABAergic synapses at the soma. (a)** Axo-somatic synapses were quantified after chronic high network activity. **(b)** Confocal images of somas and synapses from L2/3 somatosensory cortex, from P18 mice. Cells were thresholded and automatically segmented as shown in 'VGAT count'. **(c)** Density of synapses on the soma across groups. Values expressed as mean ± s.e.m. *p<0.05 **p<0.01 tested with one-way ANOVA with Sidak's posthoc comparison test. CNO control: n = 30 neurons, 3 mice; Saline control: n = 20 neurons, 3 mice; hM3Dq: n = 30 neurons, 3 mice, hM3Dq-network: n = 30 neurons, 3 mice. **(d)** Representative traces of spontaneous IPSCs (sIPSCs). **(e)** Interspike interval (ISI), **(f)** average frequency and **(g)** amplitude of sIPSCs. Values in e-g expressed as mean ± s.e.m. *p<0.05 ***p<0.001, tested with Kruskal-Wallis test followed by Dunn's multiple comparisons test. CNO control: n = 11 neurons, 3 mice; hM3Dq-network: n = 19 neurons, 5 mice, hM3Dq+: n = 13 neurons, 3 mice.

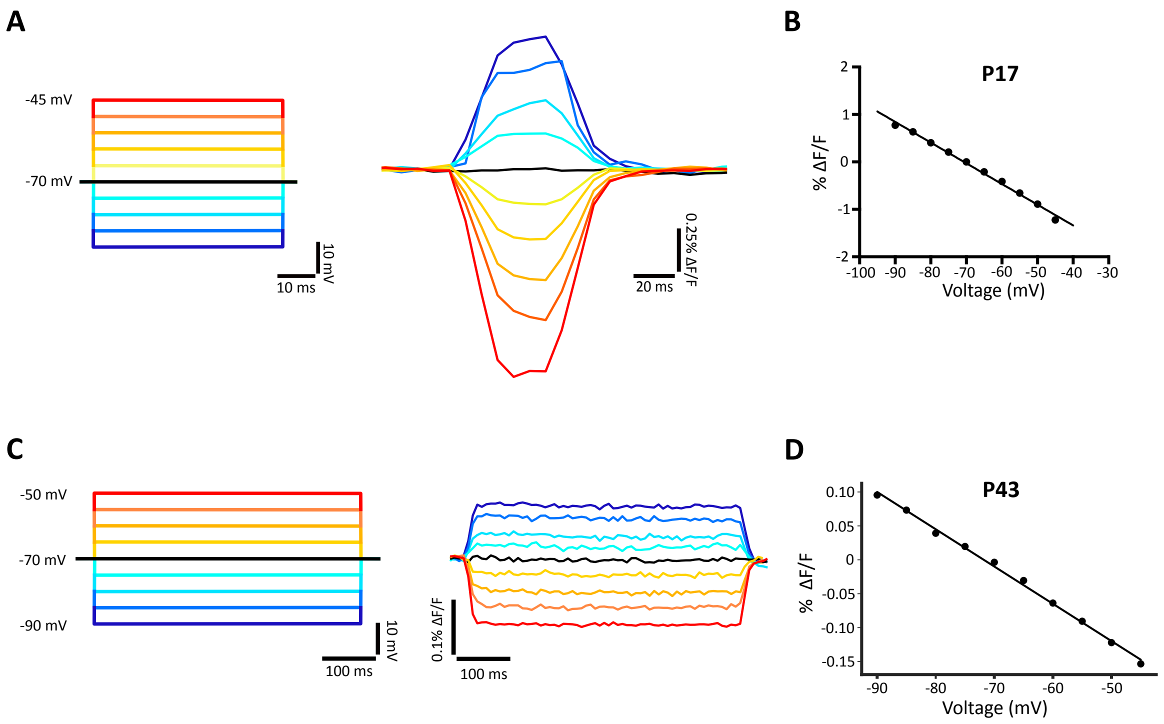

Figure S4. ***Related to Figures 3, 6 and S6.*** **Ace2N-mNeon accurately reports small changes in voltage in acute slices and maintains its sensitivity in adult mice (>P40). (a)** Representative example of a fluorometric trace in response to voltage steps during a whole cell patch-clamp experiment in a pyramidal cell expressing Ace2N-mNeon at P17. Yellow/red traces represent depolarizing voltage steps. Blue traces represent hyperpolarising voltage steps. **(b)** Maximum response (steady state) as a function of membrane voltage in the representative cell. Black line, linear regression ($p<0.0001$, r2 = 0.994). **(c)** Fluorometric traces in response to voltage steps during a whole cell patch-clamp experiment in a pyramidal cell expressing Ace2N-mNeon at P43. **(d)** Maximum response (steady state) as a function of membrane voltage. Black line, linear regression (Pearson's correlation $p<0.0001$, r2 = 0.997).

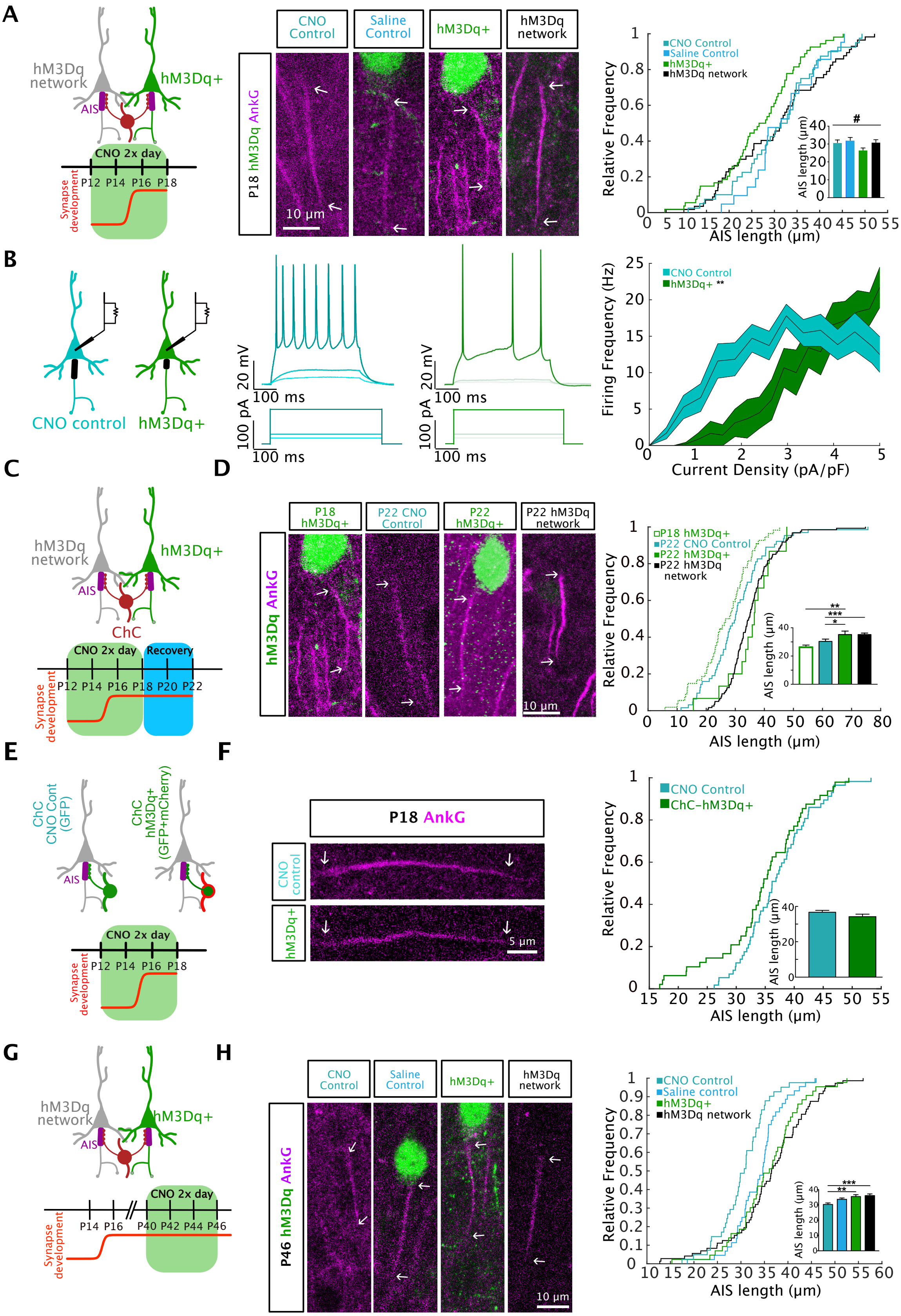

Figure S5. ***Related to Figure 2, 4, 5 and 6.*** **Pyramidal cell activity drives AIS plasticity during the period of axo-axonic synapse formation (a)** Left, timeline for chronic activation of hM3Dq in L2/3 networks with a mixed population of hM3Dq+ (green) and hM3Dq- (grey) pyramidal cells. Middle,

representative images of AISs from L2/3 somatosensory cortex from P18 mice. White arrows delimit AIS. Right, length of the AIS across treatment groups. Values expressed as mean ± s.e.m., # denotes $p<0.05$ for one-way ANOVA without reaching significance in posthoc comparisons. CNO control: n = 57 neurons, 3 mice; saline control: n = 21 neurons, 4 mice; hM3Dq+: n = 54 neurons, 4 mice; hM3Dq network: n = 40 neurons, 4 mice. **(b)** Left, CNO control (cyan) and hM3Dq+ neurons (green) were patched in whole-cell configuration to examine the intrinsic excitability properties after the treatment in (a). Middle, example firing profiles of a control (cyan) and hM3Dq+ (green) pyramidal cell to 500 ms current injections (steps shown: 10, 20 and 120 pA). Right, input output curve for each group normalised to the neuron's capacitance (current density). Centre line represents mean firing rate. Shaded area represents mean ± s.e.m. **$p<0.05$, repeated measures ANOVA. CNO control: n = 7 neurons, 2 mice; hM3Dq+: n = 8 neurons, 2 mice. **(c)** Logic of experimental design and timeline of CNO application, including recovery period. **(d)** Left, Example images of AISs from L2/3 somatosensory cortex at P22 and P18 from (a). Right, AIS length across conditions. * $p<0.05$, **$p<0.01$. ***$p<0.001$, P22 conditions tested with Kruskal-Wallis test followed by Dunn's multiple comparisons test. P18 hM3Dq+ & P22 hM3Dq+ comparison tested with unpaired student's t-test. Values expressed as mean ± s.e.m. P22 CNO control: n = 63 neurons, 2 mice; P22 hM3Dq+: n = 15 neurons, 3 mice, P22 hM3Dq-network: n = 120 neurons, 3 mice. **(e)** Experimental conditions generated by viral strategy, with ChCs expressing hM3Dq and GFP or control ChCs expressing GFP only. **(f)** Left, example images of AISs targeted by CNO control or hM3Dq+ ChCs. Right, AIS length across conditions. Differences tested with Mann-Whitney test. Values expressed as mean ± s.e.m. CNO control: n = 57 AIS, hM3Dq+: n = 48 AIS, 3 mice. **(g)** Timeline for activation of hM3Dq in L2/3 networks with a mixed population of hM3Dq+ (green) and hM3Dq- (grey) pyramidal cells in adult mice. **(h)** Left, Example images of AISs from L2/3 somatosensory cortex from P46 mice after adult treatment. Right, AIS length across conditions. *$p<0.05$, **$p<0.01$, ***$p<0.001$ tested with Kruskal-Wallis and Dunn's posthoc comparisons test. Values expressed as mean ± s.e.m. CNO control: n = 45 neurons, 3 mice; Saline control: n = 53 neurons, 5 mice, hM3Dq: n = 53 neurons, 3 mice, hM3Dq-network: n = 75 neurons, 3 mice.

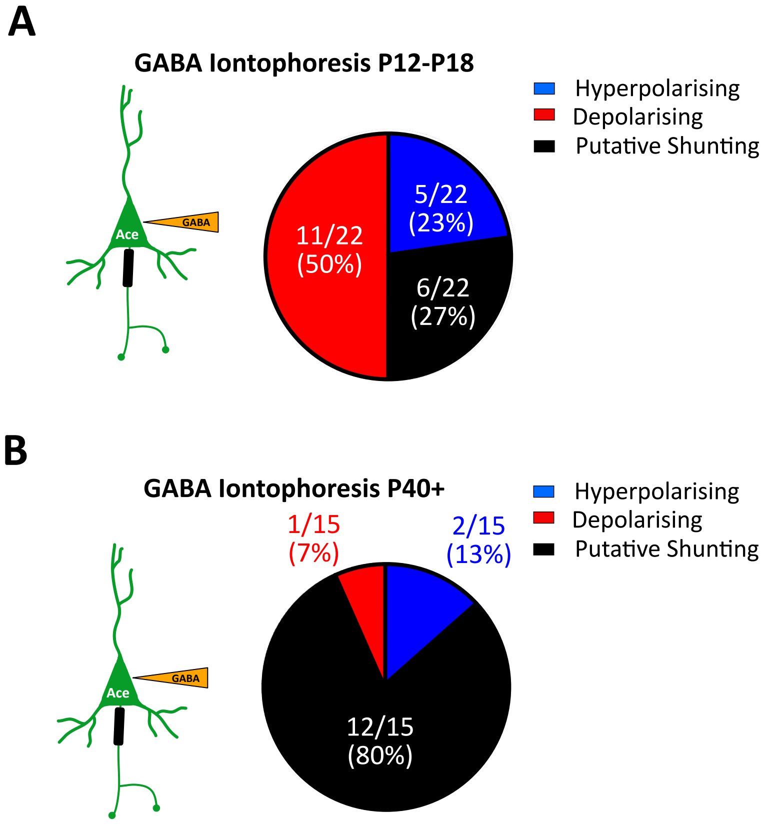

Figure S6. ***Related to Figures 3 and 6.*** **GABA polarity at the soma becomes more hyperpolarising with age.** Classification of responses into hyperpolarising (blue), depolarising (red), and putative shunting (black) for optical voltage recordings during GABA iontophoresis onto the soma of (a) peri-adolescent and (b) young adult pyramidal cells.
